# A Cross-Sectional Study of Bone Nanomechanics in Hip Fracture and Aging

**DOI:** 10.3390/life13061378

**Published:** 2023-06-13

**Authors:** Richard Stavri, Tabitha Tay, Crispin C. Wiles, Erica Di Federico, Oliver Boughton, Shaocheng Ma, Angelo Karunaratne, John H. Churchwell, Rajarshi Bhattacharya, Nicholas J. Terrill, Justin P. Cobb, Ulrich Hansen, Richard L. Abel

**Affiliations:** 1 MSk Laboratory, Department of Surgery and Cancer, Faculty of Medicine, Imperial College London, London W6 8PR, UK; 2 Warwick Medical School, University of Warwick, Coventry CV4 7AL, UK; 3 Department of Bioengineering, Faculty of Engineering, Imperial College London, London SW7 2AZ, UK; 4 Department of Mechanical Engineering, Faculty of Engineering, Imperial College London, London SW7 2AZ, UK; 5 Department of Mechanical Engineering, Faculty of Engineering, University of Moratuwa, Moratuwa 10400, Sri Lanka; 6 Department of Medical Physics and Biomedical Engineering, University College London, London WCIE 6BT, UK; 7 St. Mary’s Hospital, Northwest London Major Trauma Centre, Imperial College London, London W2 1NY, UK; 8 Diamond Light Source Ltd., Harwell Science and Innovation Campus, Didcot OX11 0DE, UK

**Keywords:** nanomechanics, biomechanics, hip fracture, aging, bone health

## Abstract

Bone mechanics is well understood at every length scale except the nano-level. We aimed to investigate the relationship between bone nanoscale and tissue-level mechanics experimentally. We tested two hypotheses: (1) nanoscale strains were lower in hip fracture patients versus controls, and (2) nanoscale mineral and fibril strains were inversely correlated with aging and fracture. A cross-sectional sample of trabecular bone sections was prepared from the proximal femora of two human donor groups (aged 44–94 years): an aging non-fracture control group (*n* = 17) and a hip-fracture group (*n* = 20). Tissue, fibril, and mineral strain were measured simultaneously using synchrotron X-ray diffraction during tensile load to failure, then compared between groups using unpaired t-tests and correlated with age using Pearson’s correlation. Controls exhibited significantly greater peak tissue, mineral, and fibril strains than the hip fracture (all *p* < 0.05). Age was associated with a decrease in peak tissue (*p* = 0.099) and mineral (*p* = 0.004) strain, but not fibril strain (*p* = 0.260). Overall, hip fracture and aging were associated with changes in the nanoscale strain that are reflected at the tissue level. Data must be interpreted within the limitations of the observational cross-sectional study design, so we propose two new hypotheses on the importance of nanomechanics. (1) Hip fracture risk is increased by low tissue strain, which can be caused by low collagen or mineral strain. (2) Age-related loss of tissue strain is dependent on the loss of mineral but not fibril strain. Novel insights into bone nano- and tissue-level mechanics could provide a platform for the development of bone health diagnostics and interventions based on failure mechanisms from the nanoscale up.

## 1. Introduction

There is a growing consensus that bone mechanical properties are influenced by the structure of the basic nanoscale building blocks of bone tissue, including collagen fibrils coated and embedded with mineral apatite [1]. Potentially, any changes in bone nanostructure and nanomechanics associated with aging could be important for fragility fractures [2,3]. Therefore, several recent reviews have stated the need to expand multiscale studies of bone tissue structure and mechanics to include the nanoscale [2,4,5,6] so that the effect of aging on bone tissue may be fully resolved [1]. The first question we must address is: *do nanoscale bone mechanics change with age?* The answer will help establish whether the intrinsic nanoscale mechanics of bone could contribute to age-related fragility fractures.

The limiting factor for investigating age-related changes in nanoscale mechanics is the technical difficulty of visualizing and quantifying the nanolevel (i.e., 10^−9^ m) structure and mechanics of bone matrix under load [7]. One of the few methods available to interrogate bone at this length scale is synchrotron small/wide angle SAXS/WAXS) X-ray diffraction [7], a state-of-the-art imaging technique that has recently enabled advances in our knowledge of bone structure [8]. X-ray diffraction can also be used to improve our knowledge of strain (i.e., deformation) and fracture mechanisms in bone because imaging can be combined with experimental mechanical testing for the simultaneous measurement of nano- and tissue-scale mechanics [9]. Consequently, bone mechanics and fracture can be measured over multiple length scales [9], allowing for a link to be made between mechanics at the nanoscale and how they exert influence on overall mechanical properties [10] at the tissue and whole bone levels.

**Figure 1:**
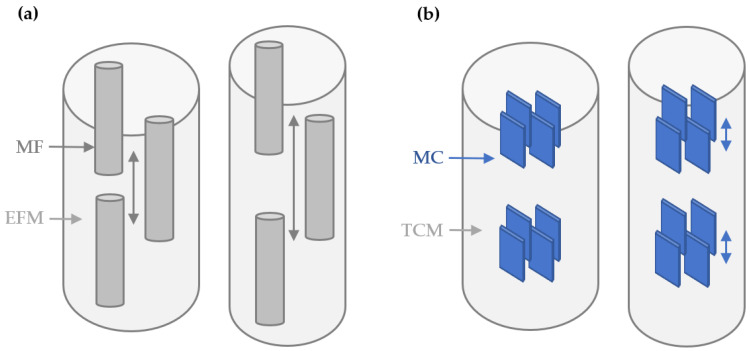
Bone nanomechanics of sliding and separation. (**a**) Bone tissue consists of mineralized fibrils (MF) surrounded by an extrafibrillar matrix (EFM). Tensile loads cause inter-fibrillar shear, then sliding and separation within the extrafibrillar matrix (grey double-headed arrows). (**b**) Each MF contains type 1 collagen molecules (TCM) with apatite crystallites (MC). Tensile loads cause intra-fibrillar MC shear (blue double-headed arrows), then sliding and separation within the collagen matrix.

Published nanomechanical studies of bone with SAXS and WAXS suggest that collagen fibrils (Figure 1a) and mineral apatite particles (Figure 1b) deform concurrently in response to tissue deformation (and vice versa). Through these nanoscale deformations, especially fibril straining [11], bone derives elasticity and probably also plasticity. Initially, the collagen fibrils and mineral particles strain co-operatively, with a transfer of tensile strains between the mineral particles via shearing (i.e., an elastic deformation) in the intervening collagen fibrils [12] facilitating elastic deformation (see Figure 1b). Subsequently, plastic (i.e., permanent) deformation occurs when the interfaces between mineral and collagen are sheared beyond the breaking point, so the mineral particles are bearing strain at a maximum and are unable to strain further. The mineral particles then begin to slide relative to each other in the collagen matrix or even start to fracture [13].

Overall, the published evidence supports the theory that these nanostructural strain and failure mechanisms, including strain transference, could be key to understanding bone mechanics at the tissue and whole bone level [12] and the effects of aging and disease. A bone’s fracture resistance is derived from structural mechanisms over a range of length scales, from the molecular scale to the microscale, and all are individually related to the nanoscale mechanics of the tissue. However, these important phenomena are poorly understood experimentally. Any data on bone nanomechanics (especially in humans) is limited to a few small studies, in part because of the difficulty in accessing synchrotron imaging facilities to conduct experiments (e.g., Diamond Light Source, European Synchrotron Radiation Facility, Argonne National Laboratory, and the Australian Synchrotron ANSTO) and the small number of groups trained to use the facilities. More research studies are needed to develop and test hypotheses about the role of nanoscale structural mechanisms in bone aging and fracture resistance.

To date, only three published studies have investigated the relationship between nanoscale bone mechanics, aging, osteoporosis, and fracture. In the first study [11], samples of human cortical bone were sectioned from the diaphyses of the humeri in two groups of middle-aged (34–41 years, *n* = 7) versus old-aged (85–99 years, *n* = 6) donors. SAXS and WAXS imaging were applied to measure nanoscale fibril and mineral strain under tensile load to failure. Older bone exhibited lower fibril (~25%) and mineral (~15%) strain than middle-aged bone. The authors concluded that fibrils became stiffer with age and absorbed less energy, while loads were transferred to the mineral less effectively. The observed loss of nanoscale strain was associated with a decline in fracture toughness measured using 3-point bending. In the second study [14], samples of human cortical bone were sectioned from the diaphyses of femora in two groups of middle-aged (34.8 ± 4.8 years, *n* = 5) versus old-aged osteoporotic (80.2 ± 9.4 years, *n* = 5) donors. SAXS imaging was applied to measure nanoscale fibril strain as a function of tissue strain under tensile load to failure. Under applied load, the fibril strain increased before plateauing, and old-aged bone exhibited a more pronounced plateauing of fibril strain and lower yield stress. The authors concluded that fibrils were less able to slide, and the tissue was less able to deform plastically. In the third study [13], samples of trabecular bone were sectioned from the femoral heads of hip-fracture patients (57–84 years, *n* = 10) versus control donors (74–94 years, *n* = 10). SAXS and WAXS imaging were applied to measure nanoscale fibril and mineral strain under tensile load to failure. In comparison to controls, the hip fracture bone exhibited significantly lower fibril and mineral strain, in combination with lower tissue strain and ultimate tensile strength. The authors concluded that aged hip fractures were associated with lower peak fibril and mineral strain because the mineral crystals fractured at lower tissue strain, leading to an earlier onset of fibril sliding. Taken together, the findings of these 3 studies suggest that aging, osteoporosis, and fracture are all associated with low fibril and/or mineral strain, which reduces the ability of bone tissue to absorb energy and reduces the resistance to cracking and fracture. However, all three studies were limited by small sample sizes and relied on simple categories of age rather than continuous analysis.

Our study aimed to investigate the relationship between bone nanoscale and tissue-level mechanics experimentally by examining clinically relevant scenarios (i.e., hip fracture and bone aging) as “tools” to manipulate the system. In a cross-sectional study, nano- and tissue-level mechanical properties were studied in trabecular bone samples from the proximal femora of two groups: aging donors that had not suffered a fracture (controls) and aging hip-fracture patients who had not received treatments for metabolic bone disease (hip-fracture). There were two objectives: to compare strains between the control and hip-fracture groups and correlate strains with age (range 44–94 years). We tested two hypotheses: (1) nanoscale strains were lower in hip-fracture patients versus controls, and (2) nanoscale mineral and fibril strains were inversely correlated with aging and fracture.

## 2. Materials and Methods

### 2.1. Donor Information

A control patient group was formed, comprising 17 unfractured cadaveric femora sourced from Vesalius Clinical Training Centre (UK). Donors were assessed to exclude any patient with a history of fractures, bone metabolic disease, or treatments. For the hip-fracture group, a series of 21 femoral heads were obtained from St Mary’s Hospital (London, UK), donated by patients who had both suffered an intracapsular hip fracture between May 2015 and February 2018 and who had not previously received treatment for low bone mineral density. As with the cadaveric controls, patient histories of metabolic bone disease and treatment were collected from patient records. Those with a history of primary bone disease or a related underlying condition that may lead to secondary bone disease, such as cancer, were excluded from the study. Subsequently, 20 specimens were included in the study, with one being excluded on account of avascular necrosis. It was not possible to obtain either dual-energy X-ray absorptiometry (DXA), fracture risk assessment tool (FRAX), or body mass index (BMI) data for any of the donors.

Demographic patient metadata, including sex and age, are presented in Table 1. The control group comprised 17 donors (9 males and 8 females; mean age = 69 years and SD 11 years). The hip-fracture group comprised 20 donors (6 males and 14 females; mean age = 79 years; SD = 6 years).

### 2.2. Sample Preparation

Cut from the primary tensile trabecular arcade of each of the femoral heads, 37 cuboidal sections were systematically extracted from the region superior to the trabecular chiasma [13,15]. The sections were each 12.0 mm in length, 2.8 mm in width, and 1.0 mm in thickness. Sample thickness was necessarily thin by design to enable full penetration of synchrotron X-rays required for accurate diffraction measurements. The ends of each specimen were embedded into 3D-printed holders with dental cement to serve as clamps during the testing, resulting in a gauge length of 6 mm. The specimens were stored at −80 °C and only removed from storage before tensile testing.

### 2.3. Tensile Testing

Synchrotron SAXS and WAXS imaging on the I22 beamline facility at Diamond Light Source [16] were combined with in situ tensile loading to simultaneously measure peak strain in the collagen fibrils, mineral apatite, and tissue. The measurement of peak fibril, mineral, and tissue strain has been described elsewhere both in detailed [13] and simple [1] formats. Peak fibril strain is the maximum fibril strain, and peak mineral strain is the maximum mineral strain (as observed in the tissue strain versus fibril and mineral strain curves, respectively). Peak fibril and mineral strain are both important measures of nanoscale deformation under load. Peak fibril strain coincides with the initiation of separation and sliding of collagen fibrils at the fibrillar interfaces (Figure 1a). Similarly, peak mineral strain coincides with the initiation of the separation and sliding of mineral crystals within the collagen matrix (Figure 1b).

Tensile testing was carried out using a customized micromechanical testing rig that was fully calibrated to I22. Tensile testing to failure was displacement-controlled with a strain rate of 0.001 s^−1^. Bone sections were defrosted for 30 min before tensile tests were conducted at room temperature. Tissue strain in each bone sample was calculated using a high-speed camera to measure the movement between two ink markers on the testing sample. The nanostrain measurements took advantage of the repeating nanoscale structure of bone. When exposed to X-rays, the collagen fibrils and mineral crystallites interact with the beam to create diffraction patterns. As the bone structure is stretched, the periodic length of the structure increases, and the consequent diffraction pattern shifts accordingly. The fibril and mineral strains were calculated from the SAXS and WAXS patterns, respectively, using DAWN software (v2.15) [17,18,19]. Briefly, collagen fibril strain during loading was measured by tracking shifts in the position of the 1st-order Bragg peak in the SAXS pattern, which arises from the characteristic ~67 nm meridional banding of intrafibrillar collagen packing. The mineral strain was calculated as the percentage change in the position of the (002) apatite peak in the WAXS pattern, which corresponds to the *c*-axis of the hip crystal lattice. For further details, see [13].

### 2.4. I22 Beam Setup and Specifications

*In situ*, micromechanical testing was performed with concurrent synchrotron X-ray scattering experiments at the I22 beamline. During testing, samples were irradiated by an X-ray beam with a wavelength of 1 Å (corresponding to an X-ray energy of 12.4 keV), and a beam cross-section of 200 × 200 µm at the sample at 2.5 s intervals with an exposure time of 0.5 s following [13]. Simultaneous SAXS and WAXS patterns were obtained at each exposure using high-speed PILATUS P3-2M and PILATUS P3-2ML detectors. The sample-detector distances were 6.852 m (SAXS) and 0.175 m (WAXS). The radiation dose did not exceed 30 kGy, which is the lower limit for avoiding tissue damage that may affect strain [20].

### 2.5. Data Analysis and Statistics

Graphs and statistical analyses were generated with GraphPad Prism 7 (San Diego, CA, USA). Data were assessed for normality by plotting histograms and were found to follow a Gaussian distribution, so parametric descriptive statistics (mean and standard deviation) and inferential statistical tests were applied. Strains were compared using unpaired t-tests. Associations between age and treatment versus strains were assessed using bivariate linear regression with a Pearson correlation coefficient of determination (*r*^2^) and a two-tailed test to determine whether the line of best fit differed from zero. The threshold for significance (alpha value) was set at 0.05. A power calculation was used to determine the *r*^2^ at which the donor sample size (*n* = 37) was powered to detect a significant difference in the gradient versus zero following [21].

## 3. Results

**Figure 2:**
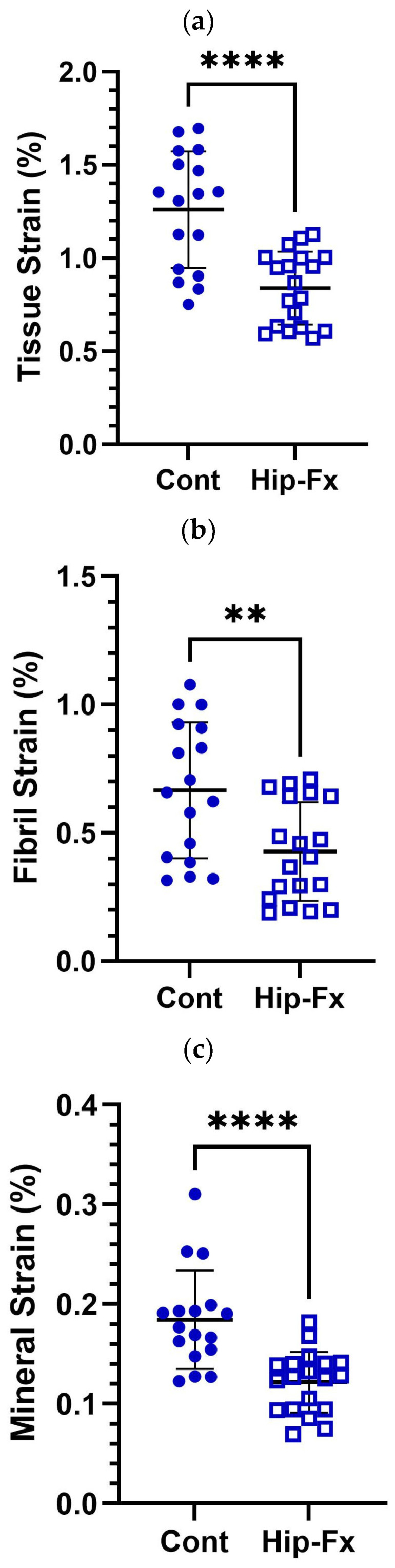
Comparison of mean strain patterns between donor groups. Non-fracture controls (Cont) exhibited significantly larger mean peak (**a**) tissue, (**b**) fibril, and (**c**) mineral strain than fracture (Hip-Fx) patients. Mean and standard deviation with unpaired *t*-test test of significance (** *p* < 0.05, **** *p* < 0.0001).

**Figure 3:**
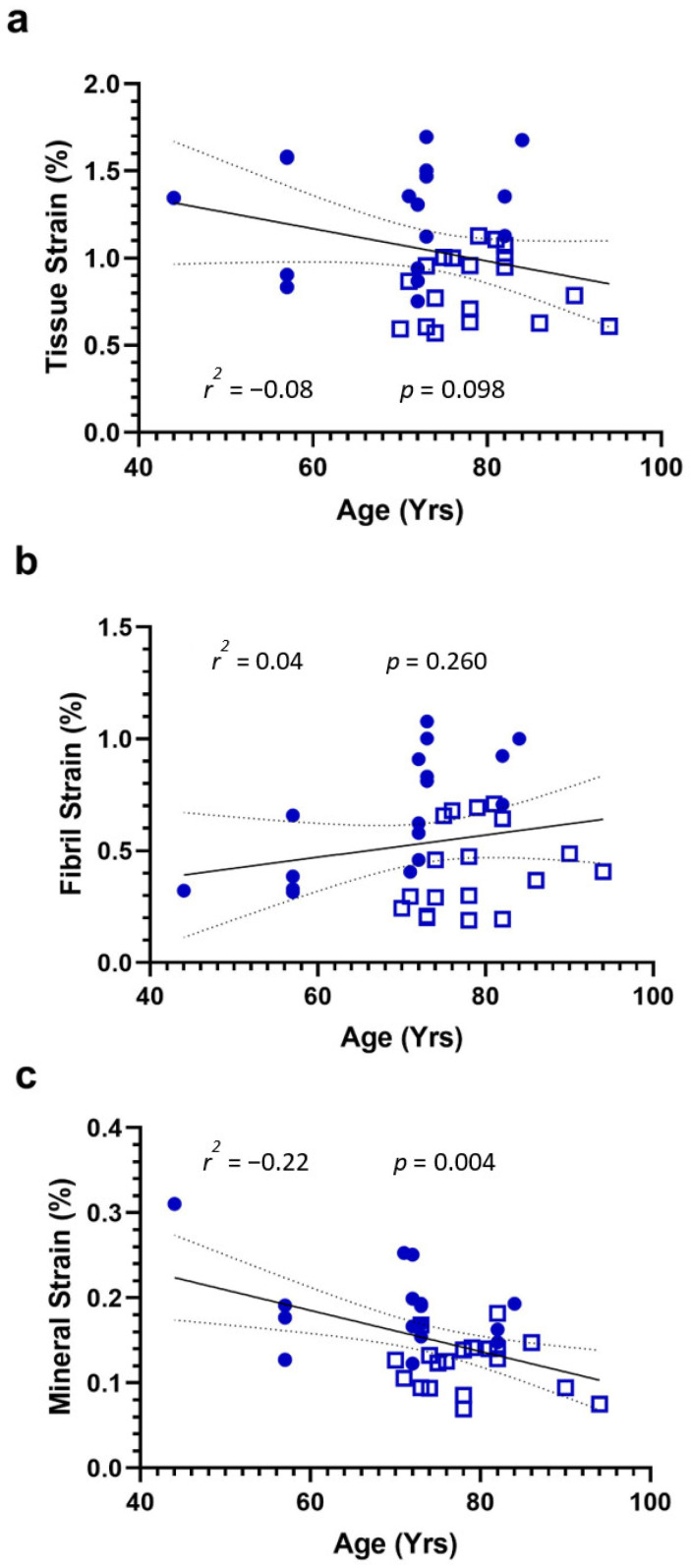
Association between nanoscale strain and age. Pearson correlation for (**a**–**c**) combining the non-fracture controls (filled blue squares) and the hip-fracture donors (open blue squares). Abbreviations denote the coefficient of determination (*r*^2^) and a significant difference versus gradient zero (*p*).

Comparisons of strain between groups are illustrated in Figure 2a–c. Controls exhibited significantly larger peak tissue (*p* < 0.0001), fibril (*p* < 0.05), and mineral strain (*p* < 0.0001) than hip-fracture donors. Regressions of strain versus age are illustrated in Figure 3a–c. For the combined control and hip-fracture groups, peak tissue strain (*r*^2^ = −0.08, *p* = 0.098) and peak mineral strain (*r*^2^ = −0.22, *p* = 0.004) were weakly and negatively correlated with age, but fibril strain was invariant with age (*r*^2^ = 0.04, *p* = 0.260). Only the negative correlation between age and peak mineral strain was significant (*p* = 0.004), but the negative correlation between age and tissue strain approached significance (*p* = 0.098). The power calculation revealed that the donor sample size *(n* = 37) was powered to determine whether the line of best fit differed significantly from zero for correlation coefficients > *r*^2^ = 0.40.

## 4. Discussion

The relationship between bone nanoscale and tissue-level mechanics was investigated experimentally by examining clinically relevant scenarios, i.e., hip fracture and bone aging. Hip fracture and aging were both associated with changes in the nanoscale strain that are reflected at the tissue level. The data reported here must be interpreted within the limitations of the observational cross-sectional study design, so in the discussion below, we consider the results and propose two new hypotheses about the role of nanomechanics in hip fracture and aging. (1) Hip fracture risk is increased by low tissue strain, which can be caused by low collagen or mineral strain. (2) Age-related loss of tissue strain is dependent on loss of mineral strain, not fibril strain. These new hypotheses need to be tested as access to human tissue banks and imaging techniques improves because new insights into nanoscale bone mechanics could provide a platform for the development of bone health diagnostics and interventions based on the material properties of bone rather than just mineral content.

### 4.1. Hip-Fractures

The data presented in Figure 2 show that, in comparison to controls, bone tissue taken from hip-fracture donors exhibited low tissue and nanoscale strain in both the collagen fibril and mineral apatite. The comparison of material strain between hip-fracture and control donors confirms the findings from our previous publication on nanoscale strain in aging fracture versus non-fracture donors [13]. Together, these studies suggest that hip fractures are associated with impaired material properties at the nanoscale. We, therefore, propose a new hypothesis that hip fracture risk is increased by low tissue strain, which can be caused by low collagen or mineral strain.

We can propose a nanostructural mechanism for tissue strain based on our observed nanoscale material strains under load. Tensile loads applied to the trabecular bone sections caused the prepared control and hip-fracture samples to deform and the mineral-fibril nanostructure to take up some of the strain. However, in tissue from hip-fracture donors, the peak mineral and fibril strains were lower than the controls, and occurred earlier, i.e., at lower tissue strains (see Figure 2a). The mineral strain observed in this study could possibly involve nanoscale fracture of the hydroxyapatite (HAp) nanocrystals, which could be an initiating event in the cracking and fracture of a bone. Nano-fractured HAp crystals might separate from the collagen and transfer strain to the fibrils, which are less stiff and can withstand greater deformation before breaking. Early shearing and separation of minerals within the collagen would reduce the amount of energy the nanoscale matrix could absorb and might therefore have contributed to the donor’s hip fractures by increasing fragility because cracks would initiate and propagate into fractures at lower applied traumatic loads. Hence, the strength of the interaction at the interface between collagen and mineral [22] could be a nanoscale fracture toughening mechanism [23,24,25,26].

The key limitation of the loading experiment and our interpretation is that the mechanics of the collagen-mineral interface must be inferred because synchrotron diffraction can only capture the collagen and mineral independently, albeit simultaneously. The interactions at the collagen-mineral interface could be compared between hip-fracture patients and controls by measuring the binding energy between collagen and minerals using imaging techniques like X-ray photoelectron spectroscopy (XPS), which measure photoelectric effects.

### 4.2. Aging

The data presented in Figure 3 show that peak tissue strain tended to decline with age (*p* = 0.098), and there was a significant age-related decline in peak mineral strain (*p* = 0.004), accompanied by a tendency for peak fibril strain to increase (*p* = 0.260). The data suggest that a loss of peak mineral strain with age contributes to a loss of tissue strain, but the loss might be offset by an increase in fibril strain. We, therefore, propose a new hypothesis that age-related loss of tissue strain is dependent on loss of mineral strain, not fibril strain.

We can propose a nanostructural mechanism for the observed patterns of variation in tissue and nanoscale strain with age. The data suggest that with advancing age, the mineral and fibril components of the matrix might shear and decouple at lower loads. In the section above, we explained that the decoupling of mineral apatite from the collagen fibril might be an initiating event in a bone fracture [1,13], so it is interesting that the ability of minerals to strain and bear load might decrease with age. The observed loss of peak mineral strain with age might contribute to a reduction in the ability of the tissue to absorb energy (Figure 3a).

The key limitation of this experiment is the observational cross-sectional study design, with all the potential selection biases that cannot be adjusted for. The limitation of the study design is especially important because our aging strain data agrees with some published studies (i.e., tissue) but not others (i.e., fibril). Many studies have reported similar reductions in peak tissue strain in human cortical tissue associated with age [27,28]. Published studies report peak tissue strains have been observed to decrease between 30 and 50% overall from middle to old age as tissue transitions from a ductile to a brittle material [29]. In this study, the reduction in peak tissue strain based on the gradient of the regression line between 44 and 96 years was about 46% (Figure 3a), at the top end of the reported range. However, published studies also report that peak fibril strains have been observed to decrease with age in cortical bone. Samples from old donors (~90 years, *n* = 4) exhibited lower fibril deformation under tensile stretching load than middle-aged donors (~40 years, *n* = 7) [11]. The result indicates that when old bone tissue is loaded with tension, the fibrils strain less before sliding and separating than in middle-aged bone. The reason for these different findings is not clear but could be related to the type of tissue examined, as Zimmerman and colleagues tested cortical bone, whereas the mechanical data presented here are from trabecular bone.

### 4.3. Crystal Size and Composition Might Affect Nanomechanics

A decrease in peak mineral strain with advancing age could occur because the nanosized hydroxyapatite mineral crystallites become less deformable (via a change in size or composition). Any changes in mineral structure or composition could promote decoupling either by putting greater stress on the interaction between the two components or by causing the interactions between collagen and mineral to become weaker, i.e., the collagen-HAp bond [22]. The effects of age on mineral size and composition are complex. The most comprehensive aging study was based on iliac crest biopsies and reported that between 0 and 90 years, mineral crystal size decreased (before slightly increasing in the oldest individuals), and the composition was altered by the replacement of phosphate with carbonate impurities [30]. Either of which could account for the decline in peak mineral strain observed here with age. Research into nanoscale mechanics needs to extend to include data on the nanostructure and composition of the mineral along with nanomechanics.

### 4.4. Limitations of Synchrotron Experiments

Limitations in the measurement of nanoscale strain using X-ray diffraction methods have been extensively detailed elsewhere [13]. The main limitation in this study is the distribution of age across the sample, with perhaps too few donors (*n* = 5) under 70 years for the significance of the correlations to be completely reliable because the under-40s might create a discontinuous distribution. Secondly, only one regression was significantly different from zero, i.e., age versus mineral (although age versus tissue strain approached significance), but this may be due to a lack of power for some of the calculations. The sample size of *n* = 37 was only sufficient to determine whether a correlation coefficient differs from zero when the coefficient of determination (*r*^2^) was greater than 0.40. However, it is important not to confuse statistical significance with practical importance, and the correlation coefficients of determination (*r*^2^) that assess performance are mostly low (e.g., *r*^2^ −0.22 to 0.04). These coefficient values are not useful for predictive purposes (which is often the case with real-world data on human anatomy and physiology, especially in cross-sectional studies), but they show some meaningful relationships in the context of understanding the contributions of nanoscale mechanics to bone fragility. Further, the observed patterns of strain variation with age do make logical sense and are broadly supported by the nano-mechanical [11,13,14] and micro-damage [7,31] data in the published literature. Certainly, the data set presented here is the best-published data set on human aging in nanoscale mechanics and, at the very least, highlights the gap in our knowledge about age-related strain transference phenomena. Nanoscale strain, including the structural mechanisms, will require close study because the skills, expertise, and knowledge acquired will help us understand the mechanisms of age-related fractures and the fracture prevention effects of treatments. For example, an ideal next step would be to study age and multiscale mechanics in cortical bone, which has a simpler microarchitecture.

## 5. Conclusions

Bone nanomechanics was investigated experimentally by studying two clinically relevant scenarios, including hip fracture and bone aging as “tools” to manipulate the system. Hip fracture and aging were both associated with changes in the nanoscale strain that are reflected at the tissue level. Two new hypotheses were formulated based on the human data. (1) Hip fracture risk is increased by low tissue strain, which can be caused by low collagen or mineral strain. (2) Age-related loss of tissue strain is dependent on loss of mineral strain, not fibril strain. More nanostructural and nanomechanical data sets are needed to improve the reliability of cross-sectional correlations, confirm the hypotheses, and estimate the size effect. Novel insights into the fundamental nanomechanics of bone tissue, including the interface between collagen and minerals, could provide a platform for the development of bone health diagnostics and interventions based on the material properties and failure mechanisms from the nanoscale up.

## Figures and Tables

**Table 1:** Demographic and structural data for two comparative groups. Aging donors that had not suffered a fracture (control) versus aging hip-fracture patients (Hip-Fx). Females (F) and males (M).

Control		Hip-Fx	
Sex	Age	Sex	Age
F	82	F	90
F	82	F	88
F	73	F	82
F	73	F	82
F	72	F	82
F	72	F	81
F	72	F	78
F	72	F	78
M	84	F	76
M	73	F	75
M	73	F	74
M	71	F	74
M	57	F	73
M	57	F	71
M	57	M	94
M	57	M	86
M	44	M	79
		M	78
		M	73
		M	70
Mean	69		79
SD	11		6

## Data Availability

The data presented in this study are available upon request from the corresponding authors.
